# SABER-Bupivacaine Reduces Postoperative Pain and Opioid Consumption After Arthroscopic Subacromial Decompression: A Randomized, Placebo-Controlled Trial

**DOI:** 10.5435/JAAOSGlobal-D-21-00287

**Published:** 2022-05-17

**Authors:** Anders Ekelund, Andrejs Peredistijs, Josef Grohs, Jon Meisner, Neil Verity, Sten Rasmussen

**Affiliations:** From the Department of Orthopaedics, Capio St Görans Hospital, Stockholm, Sweden (Dr. Ekelund); the Department of Orthopaedics, Clinic of Traumatology and Orthopaedics, Ādaži, Latvia (Dr. Peredistijs); the Department of Orthopaedics, Medical University of Vienna, Vienna, Austria (Dr. Grohs); DURECT Corporation, Cupertino, CA (Dr. Verity); Innocoll Biotherapeutics, Princeton, NJ (Dr. Meisner); and the Orthopaedic Research Unit, Aalborg University Hospital, and Department of Clinical Medicine, Aalborg University, Aalborg, Denmark (Dr. Rasmussen).

## Abstract

**Introduction:**

Shoulder arthroscopy can result in substantial postoperative pain. Sucrose acetate isobutyrate extended-release bupivacaine (SABER-Bupivacaine; trade name Posimir) is a novel depot formulation of bupivacaine designed to provide analgesia at the surgical site for up to 72 hours. The objective of this study was to evaluate the effect of SABER-Bupivacaine on pain and opioid consumption after arthroscopic subacromial decompression and to assess short-term and long-term safety.

**Methods:**

In this double-blind, placebo-controlled trial, 78 subjects were randomized in a 2:1 ratio to SABER-Bupivacaine 5 mL or SABER-placebo 5 mL injected into the subacromial space just before skin closure. Twenty-nine additional subjects were randomized on an exploratory basis to bupivacaine hydrochloride 20 mL, also injected subacromially. Subjects rated pain intensity on a 0 to 10 scale over the first 3 postoperative days and received intravenous or oral morphine for breakthrough pain. The coprimary efficacy end points were pain intensity on 90° shoulder flexion and cumulative morphine intake from 0 to 72 hours after surgery. The time to first use of opioid rescue analgesia was a secondary end point.

**Results:**

The mean (SD) pain intensity was 5.16 (1.94) for SABER-Bupivacaine and 6.43 (1.77) for placebo (*P* = 0.012). The median consumption of intravenous morphine equivalents was 4.0 mg for SABER-Bupivacaine and 12.0 mg for placebo (*P* = 0.010). The median time to first use of morphine rescue was 12.4 hours for SABER-Bupivacaine and 1.2 hours for placebo (*P* = 0.014). The corresponding values for bupivacaine hydrochloride were 5.16 (2.38), 8.0 mg, and 1.4 hours. The incidence and severity of treatment-emergent adverse events were similar for all treatment groups, and no functional or radiographic differences were noted at the 6-month follow-up.

**Discussion:**

Compared with placebo, SABER-Bupivacaine reduced pain and opioid analgesic consumption over 72 hours after arthroscopic subacromial decompression and prolonged the time to first use of opioid rescue analgesia. No safety signals were noted during the immediate postoperative period or at 6-month follow-up.

Shoulder arthroscopy is a widely performed orthopaedic procedure, with an estimated volume of 1.4 million cases worldwide in 2016.^1^ Arthroscopic subacromial decompression (ASD), either as an isolated procedure or combined with rotator cuff repair, accounted for more than 600,000 cases in the United States in 2017, according to several large outpatient and inpatient databases.^2,3,4,5^ Despite tremendous growth in the popularity of this procedure and accompanying advancements in analgesic techniques, many patients undergoing shoulder arthroscopy continue to experience substantial postsurgical pain.^6,7^ Undertreated postoperative pain can reduce patient satisfaction, prolong recovery time, increase immobility leading to clinical complications, and elevate the risk of developing chronic pain.^8,9,10,11^

Systemic opioid analgesics, still a mainstay of postoperative pain treatment, are associated with well-documented adverse effects that can delay mobilization and complicate postoperative care.^6,9,11^ Recently, concerns have also been raised about the contribution of postoperative opioid prescriptions to rising rates of opioid dependence and abuse.^12-14^ Multimodal approaches to pain management take advantage of the synergistic effect of combining different analgesic agents and administration techniques to improve the treatment of postoperative pain while also reducing the consumption of opioid analgesics and lessening their attendant risks.^9,15-17^ The role of local anesthetics in these strategies is promising but has so far been limited by their relatively short durations of action compared with the typical time course of postsurgical pain.^6,9^

Sucrose acetate isobutyrate extended-release bupivacaine (SABER-Bupivacaine; trade name Posimir) is a depot formulation of bupivacaine approved by the US Food and Drug Administration in February 2021 to produce analgesia at the surgical site for up to 72 hours after ASD. At the end of surgery, just before skin closure, the semiviscous solution is injected into the subacromial space under arthroscopic guidance. A single 5 mL dose of SABER-Bupivacaine contains 13.2% bupivacaine by volume or 660 mg of bupivacaine base, equivalent by weight to 743 mg of conventional bupivacaine hydrochloride (HCl). After administration, the formulation releases bupivacaine directly to the site of tissue injury at a mean rate of approximately 10 mg/h for 72 hours.^18^

In nonorthopaedic surgical models, clinically relevant reductions in 72-hour postoperative pain scores were observed in comparison with placebo control (inguinal hernia repair) and with active bupivacaine HCl control (laparotomy and laparoscopic cholecystectomy).^19,20^ The purpose of this study was to evaluate the effect of SABER-Bupivacaine on pain and opioid consumption after ASD and to assess the short-term and long-term safety of the formulation. The hypothesis tested was that subacromial injection of SABER-Bupivacaine would result in significantly greater postoperative analgesia and significantly less opioid consumption than placebo. An exploratory hypothesis, that SABER-Bupivacaine would show similar improvements compared with bupivacaine HCl, was also examined.

## Methods

This randomized, double-blind, placebo-controlled trial was conducted at nine sites in Austria, Denmark, Germany, Latvia, and Sweden. All subjects gave written informed consent to participate, and this study was approved by Independent Ethics Committees in each of the respective countries. Subjects were adults (aged 18 years or older) undergoing elective ASD under general anesthesia for the treatment of impingement syndrome. To reduce variability, the surgical procedure was limited to subacromial decompression using 3 or 4 standard instrument ports. Bursectomy, synovectomy, minor débridement, and resection of the coracoacromial ligament, distal clavicle, and subacromial spurs were permitted, but reconstructive procedures requiring the placement of sutures or suture anchors were not. Patients with glenohumeral pathologies or previous surgery on the affected shoulder were excluded, as were patients with connective tissue disorders or those taking analgesics or antidepressants on a regular basis. Presurgery MRI was obtained for all subjects to confirm that the rotator cuff was intact, both to ensure standardization of the procedure and as a precautionary measure to minimize potential exposure of the intra-articular cartilage to bupivacaine. Interscalene and other nerve blocks were prohibited so that serial pain assessments could be made beginning 1 hour after the administration of the study drug and any confounding effects of concurrent local anesthetic administration could be avoided.

Subjects were randomly assigned by a central computerized system in a 2:1:1 ratio to SABER-Bupivacaine 5 mL (660 mg bupivacaine base), SABER-placebo 5 mL (SABER vehicle without bupivacaine), or bupivacaine HCl 0.25% 20 mL (50 mg). The SABER-Bupivacaine 5-mL dose was chosen to allow 72 hours of bupivacaine delivery based on the known release rate of the SABER formulation. The bupivacaine HCl 50-mg dose was intended to supply a comparable quantity of bupivacaine during the 4- to 6-hour window of its expected activity. The study drug was injected into the subacromial space using a needle placed through intact skin, primarily using an anterolateral approach. Visual confirmation of proper placement was obtained using a camera in the posterior port. Subjects and investigators were blinded to study drug assignment, and investigators who conducted postoperative assessments were excluded from the operating room at the time of study drug administration to guard against accidental unblinding.

Subjects were observed in an inpatient setting for 7 days after surgery. All subjects received oral paracetamol as background treatment for the first 72 hours at the maximum recommended dosage of 4 g/d for body weight ≥66 kg and 2 g/d for body weight <66 kg. For breakthrough pain, subjects could request rescue medication, which consisted of oral morphine 10 mg at a 1-hour interval or, if unable to tolerate oral intake, intravenous (IV) morphine 2 mg at 5-minute intervals. After 72 hours, subjects were allowed paracetamol and oral morphine on an as-needed basis. Subjects recorded pain intensity (PI) on movement (flexion of the shoulder to 90°) and the use of paracetamol and opioid rescue medication in an electronic diary. PI was rated on an 11-point numerical scale that ranged from 0 (no pain) to 10 (worst pain imaginable) and was assessed at scheduled intervals after surgery. On day 0 (day of surgery), PI scores were collected at 1, 2, 4, 6, 8, and 12 hours postoperatively, and on days 1 to 7, they were assessed at 08:00, 12:00, 16:00, and 20:00.

The coprimary efficacy end points were the mean PI on movement from 1 to 72 hours after surgery (time-normalized area under the curve [nAUC_1-72_]) and cumulative consumption of opioid rescue medication (IV morphine mg equivalents) from 0 to 72 hours after surgery, both compared with placebo. The time to first use of opioid rescue medication was a secondary end point. Other end points included PI at rest, opioid-related symptom distress scale scores,^21^ eligibility for discharge based on the postanesthesia discharge scoring system,^22^ satisfaction with pain treatment on day 4, and proportion of subjects returning to work by day 14.

Safety assessments consisted of spontaneously reported and solicited adverse events (AEs), which were rated for severity, causality, and outcome; surgical wound-healing assessments; electrocardiography; clinical laboratory evaluations; vital signs; and physical examination. Only treatment-emergent adverse events (TEAEs), defined as AEs with onset after the administration of the study drug, were included in the safety analysis. Shoulder MRI and the Constant-Murley functionality assessment^23^ were conducted at baseline and at the 6-month follow-up. MRIs were centrally read by a blinded musculoskeletal radiologist after all subjects had completed the 6-month follow-up visit.

Pharmacokinetics parameters, including total and free plasma bupivacaine concentrations, were examined over 96 hours postoperatively for the first 56 subjects enrolled in the SABER-Bupivacaine and bupivacaine HCl groups. The maximum concentration (c_max_), time of maximum concentration (t_max_), total exposure (area under the plasma concentration-time curve through 96 hours [AUC_96_]), and elimination half-life (t_1/2_) were calculated.

The intention-to-treat (ITT) population was defined as all subjects randomized at the time of treatment. The safety population consisted of all randomized subjects who received at least partial administration of the study drug. The sample size calculation was based on an expected difference of 23.0 mg in total morphine consumption between the SABER-Bupivacaine and placebo arms, as observed in a previous trial of inguinal hernia repair in which the mean (SD) for SABER-Bupivacaine was 9.22 (14.7) mg and for placebo was 32.2 (50.6) mg.^19^ Enrollment of 112 subjects was estimated to be required to obtain 80% power to detect a statistically significant difference between SABER-Bupivacaine and placebo with a two-sided significance level of 0.05, assuming a 2:1:1 randomization ratio and the subsequent loss of 10% of enrolled subjects to prespecified postrandomization exclusions. Although the study plan called for the enrollment of a high-dose cohort (SABER-Bupivacaine 7.5 mL (990 mg), SABER-placebo 7.5 mL, and bupivacaine HCl 50 mg), an independent Data Review Committee subsequently deemed this exploration unnecessary, and it was canceled.

The PI on movement nAUC_1-72_ values for the treatment groups were compared using an analysis of variance model with the treatment group and country as factors. Missing pain scores were imputed by last observation carried forward for subjects discontinuing before 72 hours, first observation carried backward for missing initial pain scores, and linear interpolation for missing pain scores between two nonmissing scores. A post hoc sensitivity analysis was done using a mixed-effects model of repeated measures (MMRM) to compute the mean PI on movement over 1 to 72 hours after surgery. In this analysis, sex, site, treatment group, collection time, treatment by collection time, and treatment by sex were fixed factors; the subject was a random factor; and time was a repeating factor. A Markov covariance matrix was assumed. Because this model was capable of handling randomly missing values without bias and because no subjects discontinued this study, missing pain scores were not imputed. Total opioid rescue analgesic use from 0 to 72 hours after surgery was analyzed nonparametrically because the distribution was found to be nonnormal. Doses of oral morphine (and any nonmorphine opioids that may have been inadvertently administered) were converted to IV morphine mg equivalents using standard conversion factors. The time to first use of opioid rescue analgesia was determined by the Kaplan-Meier method, as was the proportion of subjects not using any opioid analgesia during the first 72 hours after surgery (not a prespecified end point).

To avoid concerns over multiplicity, a hypothesis to be statistically tested was defined for each of the coprimary end points and both null hypotheses (i.e., that the effect of SABER-Bupivacaine did not differ significantly from that of placebo) had to be rejected for the trial to be considered successful. No corrections for multiple testing were made for the secondary end points. Comparisons between SABER-Bupivacaine and bupivacaine HCl were designated as exploratory because this study was not powered to detect differences between these interventions.

## Results

**Figure 1:**
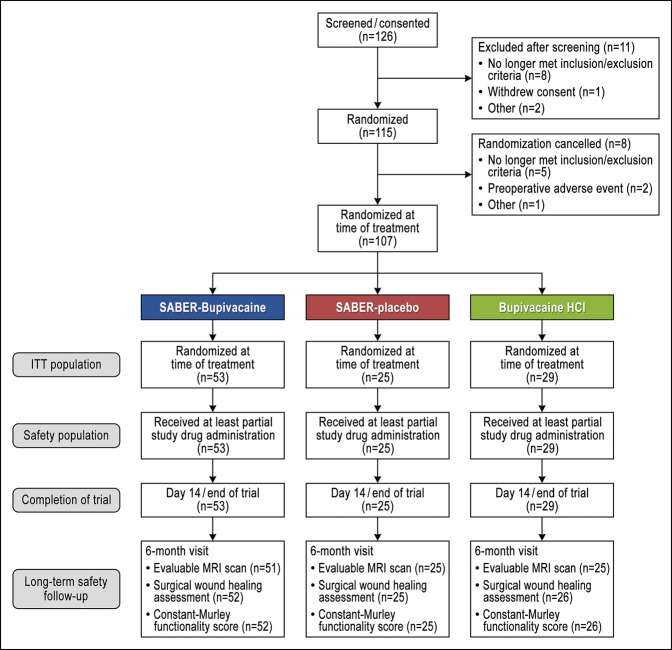
Flowchart showing study flow and subject disposition. HCl = hydrochloride, ITT = intention to treat, SABER = sucrose acetate isobutyrate extended-release.

All 107 subjects who were randomized received treatment, thereby comprising both the ITT and safety populations (Figure 1). Fifty-three subjects received SABER-Bupivacaine, 25 received SABER-placebo, and 29 received bupivacaine HCl. There were no withdrawals or discontinuations. One hundred three subjects were available for the follow-up safety evaluation at 6 months, including 101 who had evaluable MRIs. Sixty percent of the subjects were female, the mean age was 50.2 years, and nearly all were White (Table 1). No statistically significant differences were observed between treatment groups in demographic or baseline characteristics, and the specifics of the surgical procedures conducted were similar between groups (Table 2). One hour after surgery, the placebo group reported a mean (SD) PI of 8.1 (2.1) of 10, confirming that ASD can induce considerable pain in the early postoperative period. Mean 1-hour pain scores were 4.6 (2.8) for the SABER-Bupivacaine group and 6.2 (2.9) for the bupivacaine HCl group.

**Table 1:** Demographic and Baseline Characteristics

Characteristic	SABER-Bupivacaine (n = 53)	SABER-Placebo (n = 25)	Bupivacaine HCl (n = 29)	Total (N = 107)
Age (yrs)				
Mean (range)	50.1 (28-70)	48.6 (24-63)	51.6 (21-70)	50.2 (21-70)
Sex, n (%)				
Female	33 (62)	14 (56)	17 (59)	64 (60)
Male	20 (38)	11 (44)	12 (41)	43 (40)
Race, n				
Non-White	3	1	0	4
White	50	24	29	103
Body mass index (kg/m^2^)				
Mean (range)	26.8 (20.3-35.3)	25.8 (19.3-34.5)	26.7 (21.5-41.5)	25.5 (19.3-41.5)
Constant-Murley functionality score				
Mean (SD)	44.7 (12.5)	41.7 (11.7)	42.0 (11.3)	43.3 (11.9)

HCl = hydrochloride, SABER = sucrose acetate isobutyrate extended-release

**Table 2:** Surgical Procedures by the Treatment Group

Procedure, n (%)	SABER-Bupivacaine (n = 53)	SABER-Placebo (n = 25)	Bupivacaine HCl (n = 29)	Total (N = 107)
Subacromial decompression	53 (100)	25 (100)	29 (100)	107 (100)
Bursectomy	49 (92.5)	24 (96.0)	27 (93.1)	100 (93.5)
Glenohumeral joint inspection	46 (86.8)	24 (96.0)	26 (89.7)	96 (89.7)
Removal of subacromial spurs	38 (71.7)	19 (76.0)	20 (69.0)	77 (72.0)
Resection of coracoacromial ligament	32 (60.4)	18 (72.0)	16 (55.2)	66 (61.7)
Rotator cuff débridement	24 (45.3)	13 (52.0)	11 (37.9)	48 (44.9)
Synovectomy	8 (15.0)	6 (24.0)	7 (24.1)	21 (19.6)
Removal of tendon calcification	7 (13.2)	2 (8.0)	2 (6.9)	11 (10.3)
Distal clavicle excision	2 (3.8)	6 (24.0)	1 (3.4)	9 (8.4)
Articular cartilage débridement	3 (5.6)	1 (4.0)	1 (3.4)	5 (4.7)
Placement of suture anchor^a^	1 (1.9)	2 (8.0)	0 (0)	3 (2.8)
Loose body removal	0 (0)	2 (8.0)	0 (0)	2 (1.9)
Rotator cuff repair^a^	1 (1.9)	1 (4.0)	0 (0)	2 (1.9)

HCl = hydrochloride, SABER = sucrose acetate isobutyrate extended-release

a Protocol deviation (prohibited surgical procedure).

### Efficacy

**Figure 2:**
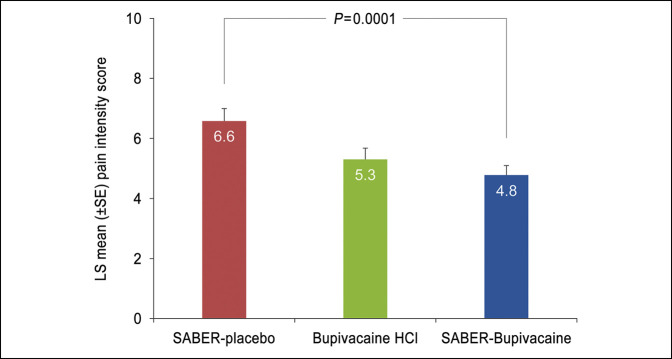
Graph showing the mean pain intensity on movement 1 to 72 hours after arthroscopic subacromial decompression. Analysis of variance comparison based on a mixed-effects model of repeated measures. HCl = hydrochloride, LS = least squares, SABER = sucrose acetate isobutyrate extended-release, SE = standard error.

PI was significantly reduced in the SABER-Bupivacaine group compared with the placebo group for the 1- to 72-hour postoperative period. The mean (SD) nAUC_1-72_ was 5.16 (1.94) for SABER-Bupivacaine and 6.43 (1.77) for placebo (*P* = 0.012). For subjects receiving bupivacaine HCl, the mean (SD) nAUC_1-72_ was 5.16 (2.38). Post hoc analysis of the PI data using the more efficient MMRM computation yielded results consistent with the prespecified nAUC_1-72_ analysis: The least squares mean (SE) PI for 1 to 72 hours was 4.8 (0.32) for SABER-Bupivacaine, 6.6 (0.42) for placebo (*P* = 0.0001), and 5.3 (0.38) for bupivacaine HCl (Figure 2).

**Figure 3:**
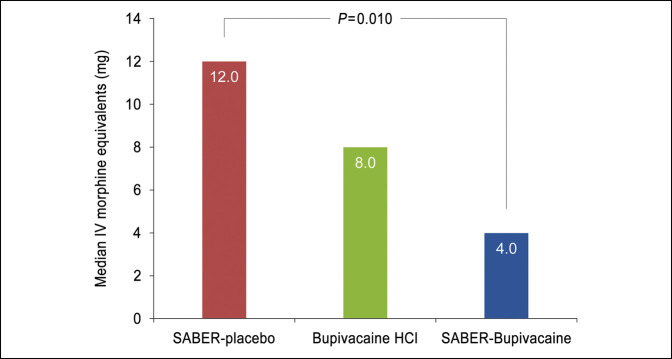
Graph showing the median cumulative IV morphine-equivalent opioid analgesic use in mg 0 to 72 hours after surgery. Comparison based on Hodges-Lehmann estimates for median difference and the Wilcoxon rank-sum test. HCl = hydrochloride, IV = intravenous, SABER = sucrose acetate isobutyrate extended-release.

**Figure 4:**
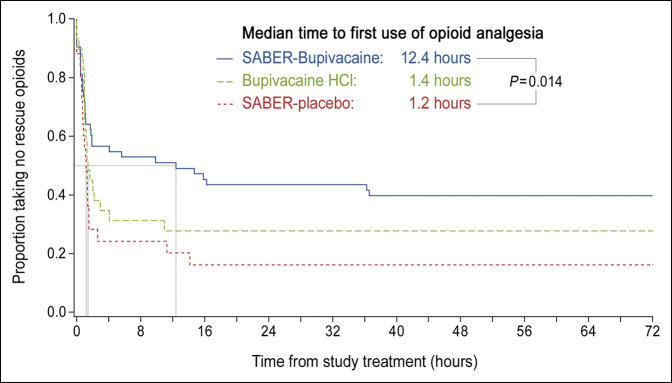
Graph showing the time to first use of opioid rescue medication after arthroscopic subacromial decompression. Comparison based on Kaplan-Meier analysis and the log-rank test. HCl = hydrochloride, SABER = sucrose acetate isobutyrate extended-release.

**Figure 5:**
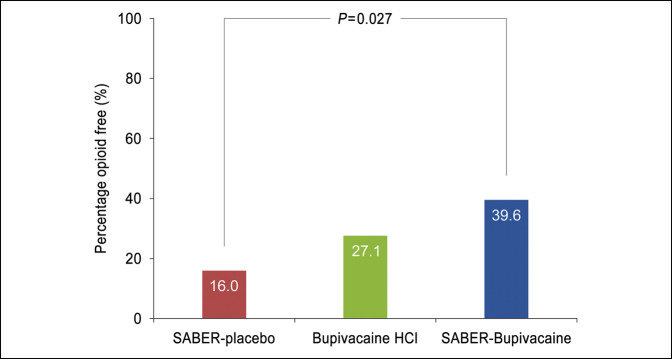
Graph showing the percentage of opioid-free subjects during the initial 72 hours after surgery. Comparison based on the Cochran-Mantel-Haenszel test. HCl = hydrochloride, SABER = sucrose acetate isobutyrate extended-release.

The total consumption of opioid rescue medication in IV morphine equivalents over the first 72 hours after surgery, the coprimary end point, was significantly reduced in subjects treated with SABER-Bupivacaine compared with placebo: The median was 4.0 mg for SABER-Bupivacaine and 12.0 mg for placebo (*P* = 0.010). For bupivacaine HCl, the median consumption was 8.0 mg (Figure 3). The time to first use of opioid rescue medication after surgery was significantly prolonged in the SABER-Bupivacaine group compared with the placebo group: The median (SE) time to first opioid analgesic use was 12.4 (8.86) hours for SABER-Bupivacaine and 1.2 (0.12) hours for placebo (*P* = 0.014). For bupivacaine HCl, the time to first use was 1.4 (0.45) hours (Figure 4). The percentage of subjects who remained opioid-free during the first 72 hours after surgery (post hoc analysis) was also significantly greater in the SABER-Bupivacaine group than the placebo group: 39.6% vs 16.0% (*P* = 0.027), respectively. The percentage abstaining from opioids in the bupivacaine HCl group was 27.6% (Figure 5).

Of the remaining secondary efficacy end points, only PI at rest showed a significant difference between treatment groups, with a mean (SD) nAUC_1-72_ of 2.50 (1.34) for SABER-Bupivacaine and 3.43 (2.05) for placebo (*P* = 0.021). The others revealed no notable differences.

### Safety

A total of 65 TEAEs were reported among 37 subjects. No subject withdrew from the trial because of a TEAE. The overall incidence of TEAEs was similar between treatment groups: 30.2% for SABER-Bupivacaine, 40.0% for SABER-placebo, and 37.9% for bupivacaine HCl (Table 3). Nine subjects (8.4%) reported a total of 10 TEAEs that were considered related to treatment: 5 subjects (9.4%) in the SABER-Bupivacaine group, 2 (8.0%) in the SABER-placebo group, and 2 (6.9%) in the bupivacaine HCl group. Most TEAEs in all treatment groups were mild or moderate in severity.

**Table 3:** TEAEs Occurring in 3 or More Subjects (>2% of the Safety Population) Through Postoperative Day 14

Factor	SABER-Bupivacaine (n = 53)	SABER-Placebo (n = 25)	Bupivacaine HCl (n = 29)	Total (N = 107)
All TEAEs, n (%)	16 (30.2)	10 (40.0)	11 (37.9)	37 (34.6)
TEAEs by primary system organ class, n				
Nervous system disorders	5	2	4	11
Headache	3	1	1	5
Investigations	5	2	2	9
Elevated alanine aminotransferase	1	2	0	3
Gastrointestinal disorders	2	3	1	6
Nausea	1	3	1	5
Musculoskeletal and connective tissue disorders	3	1	2	6
Musculoskeletal pain	2	1	2	5
Cardiac disorders	1	2	3	6
Skin and subcutaneous tissue disorders	2	2	2	6
Injury and procedural complications	3	1	0	4
General disorders and administration site conditions	1	2	0	3
Respiratory, thoracic, and mediastinal disorders	1	0	2	3

HCl = hydrochloride, SABER = sucrose acetate isobutyrate extended-release, TEAE = treatment-emergent adverse event

There were no deaths during this study. Of 6 serious AEs reported, 2 occurred during the immediate 2-week postsurgical follow-up period. One (intolerance to tramadol, a protocol-prohibited analgesic) occurred in the SABER-placebo group and the other (tongue paralysis) occurred in the bupivacaine HCl group. Neither was considered related to study drug administration.

No notable differences were observed between groups in clinical laboratory evaluations, vital signs, or surgical wound healing assessments conducted during the first 2 weeks postoperatively. Electrocardiographic studies, recorded at baseline and at 1, 4, 8, 12, 24, 36, 48, and 72 hours after surgery, revealed no clinically meaningful differences between groups. Corrected QT intervals were slightly longer at 1 hour than at baseline in both the SABER-Bupivacaine and bupivacaine HCl groups (+7 msec and +8 msec, repectively) and then returned gradually to their pretreatment values. A corrected QT interval ≥480 msec was not seen in any group.

At the 6-month follow-up visit, 101 of the 107 randomized subjects had evaluable MRI scans and 103 subjects were assessed for surgical wound healing. Other than findings attributable to the surgical procedure itself, shoulder MRI evaluations were largely unchanged from baseline. No meaningful differences were observed between treatment groups, and no safety concerns were identified from MRI results or surgical site evaluations. No subject demonstrated evidence of new-onset cartilage loss or chondrolysis, based on history, physical examination, and MRI evaluation. Constant-Murley functionality scores increased from baseline to 6 months in all 3 treatment groups, with SABER-Bupivacaine subjects (n = 52) improving from a mean (SD) of 44.7 (12.3) to 61.6 (15.1), SABER-placebo subjects (n = 25) from 41.7 (11.4) to 63.2 (12.1), and bupivacaine HCl subjects (n = 26) from 42.0 (11.1) to 65.6 (6.6).

### Pharmacokinetics

**Figure 6:**
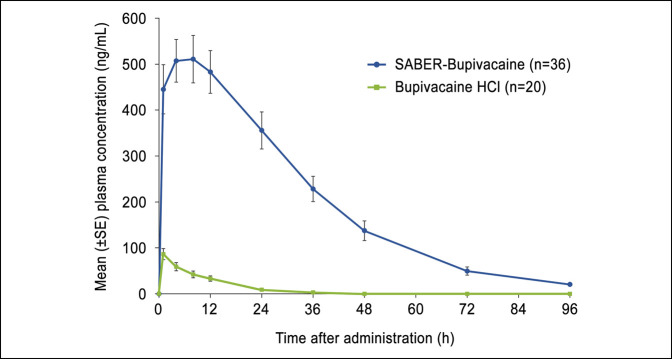
Graph showing the plasma total bupivacaine concentration 0 to 96 hours after administration. HCl = hydrochloride, SABER = sucrose acetate isobutyrate extended-release, SE = standard error.

Plasma total bupivacaine concentrations were higher at all measured time points in the SABER-Bupivacaine group (n = 36) than the bupivacaine HCl group (n = 20) (Figure 6), leading to a greater mean AUC_96_ (14,980 vs 686 ng·h·mL^−1^, respectively) and a greater c_max_ (500 [range 70 to 1,320] ng/mL vs 73 [range 8 to 195] ng/mL, respectively). The peak plasma free bupivacaine concentration was also higher in the SABER-Bupivacaine group (mean 29 [range 3 to 74] ng/mL) than the bupivacaine HCl group (mean 4 [range <1 to 10] ng/mL). The median t_max_ for SABER-Bupivacaine (5.94 hours) was delayed relative to that of bupivacaine HCl (1.03 hours), reflecting the extended-release nature of the depot formulation.

## Discussion

In this study, SABER-Bupivacaine administered as a single 5-mL injection into the subacromial space at the end of surgery significantly reduced pain on movement over 72 hours postoperatively compared with placebo. It also significantly reduced the use of opioid analgesics and prolonged the time to first use of opioid rescue medication, both in comparison with placebo. Pain at rest was significantly diminished as well with SABER-Bupivacaine treatment; however, pain on movement, defined in this study as shoulder flexion to 90°, was designated the primary pain end point because of its relevance to patient recovery and return of function and its better ability to discriminate between the effects of different analgesic interventions.^24,25^

A post hoc MMRM analysis of 72-hour PI data was consistent with the prespecified nAUC evaluation, lending additional statistical support to the observed analgesic effect of SABER-Bupivacaine. The MMRM methodology is well suited to the analysis of serial measurements having both fixed and random characteristics and offers improved statistical power compared with the prespecified AUC analysis.^26,27^

Although distal clavicle excision was more prevalent in the placebo group (24%) than the SABER-Bupivacaine group (3.8%), a sensitivity analysis showed that this imbalance had no effect on relative pain scores.

As demonstrated in this study, ASD can be associated with considerable pain during the early postoperative period. The mean 72-hour pain score for SABER-Bupivacaine was reduced by 20% (absolute reduction 1.27 on a 0 to 10 scale) relative to placebo, even in the presence of background paracetamol treatment, and it was reduced by 27% (absolute reduction 1.80) in the MMRM sensitivity analysis. These figures are consistent with the results of several previous studies in which bupivacaine or ropivacaine was infused into the subacromial space using an indwelling catheter for 24 to 48 hours after ASD.^8,28,29^ Although minimum meaningful pain reduction thresholds have not been established for acute postsurgical pain, the literature on chronic pain management suggests that a 20% to 30% reduction in PI score can be considered clinically meaningful, although this value is typically calculated in comparison with pretreatment baseline.^30-32^ Baseline postoperative pain measurements were not obtained in this study because the interventional therapies were administered during surgery while subjects were anesthetized.

No notable difference in pain scores was observed in the exploratory comparison between SABER-Bupivacaine and bupivacaine HCl, a result that may be partly explained by the small size of this study, which was powered for the primary comparison between SABER-Bupivacaine and placebo. The respective effects of background and rescue analgesia may also have contributed to this outcome. First, the universal administration of paracetamol, although reasonable from a clinical practice standpoint, may have reduced the overall pain signal, thereby blunting any difference in analgesic effect between SABER-Bupivacaine and bupivacaine HCl. Second, the median consumption of opioid rescue medication was twice as high in the bupivacaine HCl group as the SABER-Bupivacaine group, which may have led to relatively lower pain scores in this group or, conversely, indicated that pain had been underreported. Finally, the possibility that immediate-release bupivacaine HCl, for reasons unique to this study protocol or population, produced an analgesic effect considerably prolonged relative to its expected duration cannot be ruled out without additional study.

The reduced opioid consumption that accompanied SABER-Bupivacaine treatment supports the contention that the observed pain relief was clinically meaningful. The median time to the first request for opioid rescue analgesia was delayed by 11.2 hours relative to placebo and 11 hours relative to bupivacaine HCl. There was a two-thirds reduction in the overall opioid analgesic use during the first 3 days after surgery compared with placebo, and the proportion of subjects who were opioid-free during this period was 2.5 times higher. The combination of reduced pain and opioid use is consistent with the goals of multimodal pain management. Sixty-two percent of subjects treated with SABER-Bupivacaine took the equivalent of one 10-mg morphine tablet or less during the initial 72-hour postoperative period, with nearly two-thirds of that group requiring no opioid treatment at all. By contrast, just 28% of placebo subjects and 45% of bupivacaine HCl subjects were able to limit their oral equivalent morphine use to 10 mg or less, suggesting that SABER-Bupivacaine could reduce the need for routine prescribing of opioids after ASD.

SABER-Bupivacaine forms an in situ depot that releases local anesthetic to the surgical site for 72 hours after administration, markedly prolonging bupivacaine activity without the need for an external catheter or pump. Pharmacokinetics data collected in this study show a sustained 3-day exposure to bupivacaine with no initial burst and maximum plasma levels well within safety limits for the duration of therapy.^33-35^ In some soft-tissue surgery studies (laparotomy and laparoscopically assisted colectomy), the plasma t_max_ occurred as late as 48 hours after SABER-Bupivacaine administration,^18^ suggesting that the relative vascularity of the subacromial space may have hastened the uptake of bupivacaine into the systemic circulation. Because the activity of bupivacaine is considered to be predominantly local, a meaningful correlation between plasma bupivacaine concentration and analgesia at the surgical site cannot be inferred.

SABER-Bupivacaine was safe and well tolerated during the immediate postoperative period and raised no long-term safety concerns at the 6-month follow-up. Signs of systemic bupivacaine toxicity, including central nervous system effects and cardiac arrhythmias, were not detected in any group during the initial 3- to 7-day evaluation period nor were imbalances in TEAEs that could be attributed to the components of the SABER formulation.

No evidence of chondrolysis or new-onset cartilage loss was found at the 6-month evaluation either radiographically or by physical examination, and the surgical incisions healed as expected in all subjects. Degeneration of joint cartilage has been observed after prolonged, high-flow infusion of bupivacaine HCI directly into the glenohumeral joint but has not been associated with infusion into the subacromial space.^6,36,37^ Nonetheless, to provide an extra margin of safety, all enrolled subjects were required to have intact rotator cuffs, and both SABER-Bupivacaine and bupivacaine HCl were injected into the subacromial space under direct arthroscopic visualization to ensure accurate placement.

This study had several limitations. As previously noted, it was not powered to detect differences between the investigational drug and the active control bupivacaine HCl because this was not the study's primary objective. The study population, although otherwise representative of the surgical population for this procedure, included few non-White subjects. This study was restricted to individuals with intact rotator cuffs, which reduced variability but also limited generalizability. Finally, interscalene block was not used in this trial so that the early analgesic activity of the study drug could be accurately assessed and any confounding effects of additional local anesthetic administration could be avoided. For that reason, the concurrent use of SABER-Bupivacaine and interscalene block would require additional study.

## Conclusions

Compared with placebo, SABER-Bupivacaine reduced pain and opioid analgesic consumption over 72 hours after arthroscopic subacromial decompression and prolonged the time to first use of opioid rescue analgesia. No safety signals were noted during the immediate postoperative period or at 6-month follow-up.
